# The efficacy of gastric aspiration in reducing postoperative vomiting after oral and maxillofacial surgery: A meta-analysis

**DOI:** 10.1097/MD.0000000000037106

**Published:** 2024-02-16

**Authors:** Xushu Zhang, Xiaojuan Xie, Min Shi, Yao Yao, Zhen Feng, Jian Yang, Tao Guo

**Affiliations:** a School of Medicine, Huanggang Polytechnic College, Huanggang 438002, China; b Department of Pathophysiology, School of Basic Medical Sciences, Weifang Medical University, Weifang 261053, China; c School of Nursing, Huanggang Polytechnic College, Huanggang 438002, China; d Affiliated Hospital of Huanggang Polytechnic College, Huanggang 438021, China.

**Keywords:** gastric aspiration, oral and maxillofacial surgery, postoperative vomiting

## Abstract

**Background:**

Gastric aspiration is applied in oral and maxillofacial procedures to reduce postoperative vomiting (POV), yet its clinical benefit remains largely uncertain. Our study aimed to determine the role of gastric aspiration in the amelioration of POV by a meta-analysis.

**Methods:**

With adherence to the Preferred Reporting Items for Systematic Reviews and Meta-Analyses (PRISMA) guidelines, global recognized databases, including PubMed, Embase, and Cochrane Central, were searched to obtain randomized controlled trials (RCTs) investigating the effects of gastric aspiration in oral and maxillofacial surgery. The incidence and the number of episodes of POV and the frequency of rescue antiemetic use were extracted as parametric data for pooled estimation. Funnel plots and Egger’s test were utilized to assess bias. The recommendation of evidence was rated by the Grading of Recommendations Assessment, Development, and Evaluation (GRADE) system.

**Results:**

After detailed evaluation, 5 RCTs containing 274 participants were eventually included. The results of pooled estimation indicated that gastric aspiration could not reduce the incidence of POV (risk ratio [95% CI] = 0.94 [0.73, 1.21], *P* = .621), the number of episodes of POV (standard mean difference [95% CI] = −0.13 [−0.45, 0.19], *P* = .431) or the frequency of rescue antiemetic use (RR [95% CI] = 0.86 [0.49, 1.52], *P* = .609). No publication bias was detected by the funnel plot and Egger test. The overall recommendation of evidence was rated low regarding each outcome.

**Conclusion:**

Based on current evidence, gastric aspiration is not recommended for oral and maxillofacial surgery. Meanwhile, more large-scale high-quality RCTs are needed.

## 1. Introduction

Postoperative vomiting (POV) is one of the most common adverse events after anesthetic-surgical procedures.^[1,2]^ It may occur in up to 30% of cases in the first postoperative 24 hours, resulting in great discomfort for patients.^[1,3]^ In maxillofacial surgery, such as pediatric tonsillectomy, more than half of patients may have postoperative vomiting.^[4,5]^ Even among patients who underwent orthognathic surgeries, the morbidity rate of postoperative emesis was as high as 40%.^[6]^ Despite being associated with a higher rate of postoperative vomiting, oral and maxillofacial procedures are still widely conducted.^[7–9]^ Postoperative emesis increases the possibility of surgical wound bleeding, facial edema, hypesthesia, dehydration and electrolyte imbalance, which may prolong hospital stays and result in another unanticipated hospital admission.^[3,6,10,11]^ Therefore, emesis decreases the life quality and makes postsurgical care difficult after oral and maxillofacial surgeries.^[12,13]^ Postoperative vomiting, especially persistent vomiting, may simultaneously reveal significant impacts on financial and medical aspects.^[14,15]^

The mechanism of POV is complicated and is not currently completely understood. To minimize the possibility of POV, multimodal protocols were carried out for clinical referral, and surgical and anesthetic factors were considered undercontrol factors to reduce the risk of vomiting.^[16–18]^ Considering the strong association of intraoperative swallowed blood and POV, gastric aspiration was gradually applied to avoid POV based on reducing the stimulation of gastric contents and pressure by aspirating surgical foreign object debris and original stomach contents.^[3,6,11]^ Its clinical efficacy was apparently supported by some clinical studies with respect to the reduction of POV after oral and maxillofacial procedures.^[19]^ However, as the first traceable randomized controlled trial (RCT), Jones et al^[20]^ denied the clinical efficacy of gastric aspiration in reducing POV. Since then, multiple clinical trials and observations have been conducted to identify the pros and cons of gastric aspiration for oral and maxillofacial operations, but debates have remained until now.

The current study aimed to perform a meta-analysis to determine the effects of gastric aspiration on the incidence of POV after oral and maxillofacial surgeries. This study may provide relative evidence for future clinical guidelines and new research directions.

## 2. Methods

### 2.1. Retrieving and obtaining literature

The current meta-analysis was performed with the guidelines of the Preferred Reporting Items for Systematic Reviews and Meta-Analyses (PRISMA) 2020 statement.^[21]^ It does not require approval from the ethics committee since this is an meta-analysis. We preregistered this meta-analysis online before conducting data analysis at the INPLASY database with ID INPLASY202320016. Global-recognized electronic databases, including PubMed, Embase, and Cochrane Central, were retrieved by combining Medical Subject Headings items to address RCTs investigating the effects of gastric aspiration on reducing postoperative vomiting in patients who underwent oral and maxillofacial surgeries (an example of the retrieval strategy in PubMed is presented in Supplementary Table S1, Supplemental Digital Content 1, http://links.lww.com/MD/L349). Initially, addressed titles and abstracts were browsed for potential eligibility. Full texts were read in detail for consideration for final inclusion. No limitation of publication time was set, but full English text must be traced if the study was eligible for meta-analysis.

### 2.2. Inclusion and exclusion criteria

The trials were considered for inclusion if they met the following criteria: (1) randomized controlled trial, (2) trials investigating the effects on reducing vomiting in oral and maxillofacial surgeries, (3) descriptions of postoperative vomiting as the main outcome, and (4) full English text.

The following criteria were regarded as exclusion criteria: (1) nonRCTs, (2) investigations on other surgeries, (3) trials with insufficient raw data, (4) no full English text, and (5) irrelevant studies, reviews, comments and editorials.

### 2.3. Quality evaluation of methodological process and evidence

All included trials were assessed by the Jadad scoring system (ranging from 0–5 points) to determine their quality regarding methodological process by rating as high-quality (3–5 points) or low-quality (0–2 points).^[22]^ The risk of bias for each included trial was also conducted according to the Cochrane Risk of Bias assessment tool.^[23]^ Six evaluation criteria, namely, random sequencing generation, allocation concealment, blinding of participation, blinding of outcome evaluation, incomplete outcome data and selective reporting, were utilized to evaluate whether there was bias risk in each trial. Considering the quality of design and process and risk of bias, the recommendation of evidence was conducted based on 5 factors that may lower the quality of the evidence, including design limitations, inconsistent findings, indirect evidence, inaccuracy, and publication bias, to rate the recommendation of evidence regarding each outcome by the Grading of Recommendations Assessment, Development and Evaluation (GRADE) system.^[24]^ About two principle investigators individually completed all the abovementioned assessments, and any conflicting ratings were resolved by discussion among all authors.

### 2.4. Data extraction and estimation

To assess the clinical efficacy of gastric aspiration on reducing postoperative emesis, we selected the incidence and number of episodes of postoperative vomiting and frequency of rescue antiemetics as the outcomes for pooling estimation. The included raw data of the incidence of emesis and application of rescue antiemetics were presented as dichotomous variables, which were incidence with sample size in each group. Episode of vomiting was presented as the mean with standard deviation for analysis. The relative parametric data were independently extracted by the above 2 investigators, and all the extracted data were pooled and estimated by meta-analysis.

### 2.5. Statistical analysis

For the calculation of dichotomous variables regarding selected parameters, the risk ratio (RR) and its 95% confidence interval (CI) based on quantitative analysis were the final outcome to present the effects of gastric aspiration on the risk of emesis and the possibility of antiemetics use. The fluctuation of total episodes of vomiting between the gastric aspiration group and the control group is presented as the standard mean difference (SMD) with the associated 95% CI. Heterogeneity was calculated with the *I*^2^ test ranging from 0 to 100%. A random-effects model was applied if high heterogeneity (>50%) was detected. For those results with low heterogeneity (<50%), outcomes were calculated based on a fixed-effects model. Additionally, funnel plots and Egger’s test were used to assess the potential bias, and STATA software (version 15.0) was applied for statistical manipulation.

## 3. Results

### 3.1. Summary and characteristics of the included studies

**Figure 1.**
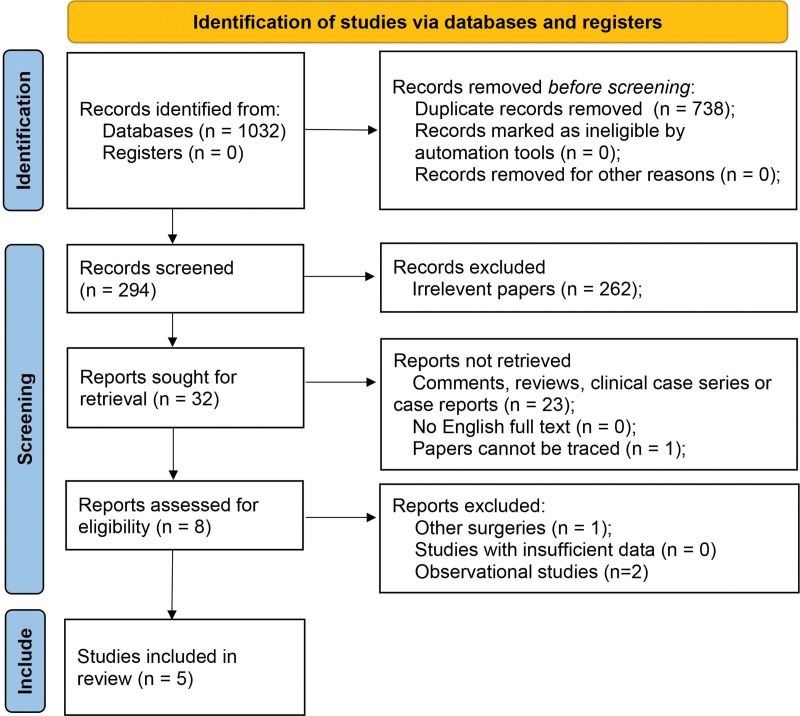
Flow diagram of the process of selecting studies for the current meta-analysis.

After initial retrieval, 1032 records were first addressed, and 294 of them were reserved after removing duplicates. After detailed evaluation of the full texts, 5 RCTs^[20,25–28]^ containing 274 patients were eventually included in the final meta-analysis (Fig. 1). All the included trials were 2-arm single center RCTs, and 4 of them^[20,25,26,28]^ were double-blinded. Three investigations reported tonsillectomy,^[20,25,27]^ and the other 2 trials^[26,28]^ were based on orthognathic surgeries (Table 1).

**Table 1:** Characteristics of included studies.

Author	Year	Region	Study design	Sample size	Surgical procedure	Prevention strategy	Observation time	Outcomes
Chukudebelu	2010	Ireland	2-arm, single center, double-blinded RCT	39	-Tonsillectomy	At the completion of operations, a gauge nasogastric tube was passed into the stomach for aspiration.	48 h	Incidence of vomiting; application of antiemetics
					-Tonsillectomy + Adenotonsillectomy			
Jesus	2022	Brazil	2-arm, single center, double-blinded RCT	83	Orthognathic surgery:	A number 18 gastric tube was inserted orally and the gastric contents were removed by aspiration.	24 h	incidence of vomiting;
					-Bimaxillary osteotomies + mentoplasty			
					-Bimaxillary osteotomies			
					-Turbinectomy			
Jones	2011	USA	2-arm, single center, double-blinded RCT	74	-Tonsillectomy	An orogastric tube was placed postoperatively under direct visualization, and the gastric contents were aspirated prior to emergence from anesthesia.	24 h	Incidence of vomiting; episodes of vomiting; application of antiemetics
					-Tonsillectomy + Adenotonsillectomy			
Muhammad	2012	Pakistan	2-arm, single center, RCT	54	Tonsillectomy	An orogastric tube was placed postoperatively under direct visualization, and the gastric contents were aspirated prior to emergence from anesthesia.	24 h	Incidence of vomiting; Episodes of vomiting; Application of antiemetics
Schmitt	2017	Brazil	2-arm, single center, double-blinded RCT	24	Combined orthognathic surgery including:	A gastric tube (number 18 or 20) was inserted orally or nasally, gastric contents were removed before patients were woken and extubated.	24 h	Incidence of vomiting; Episodes of vomiting
					-Le Fort I osteotomy			
					-Saggital Split osteotomy			
					-Genioplasty			
					-Turbinectomy			

### 3.2. Quality assessment of methodological process and bias

**Figure 2.**
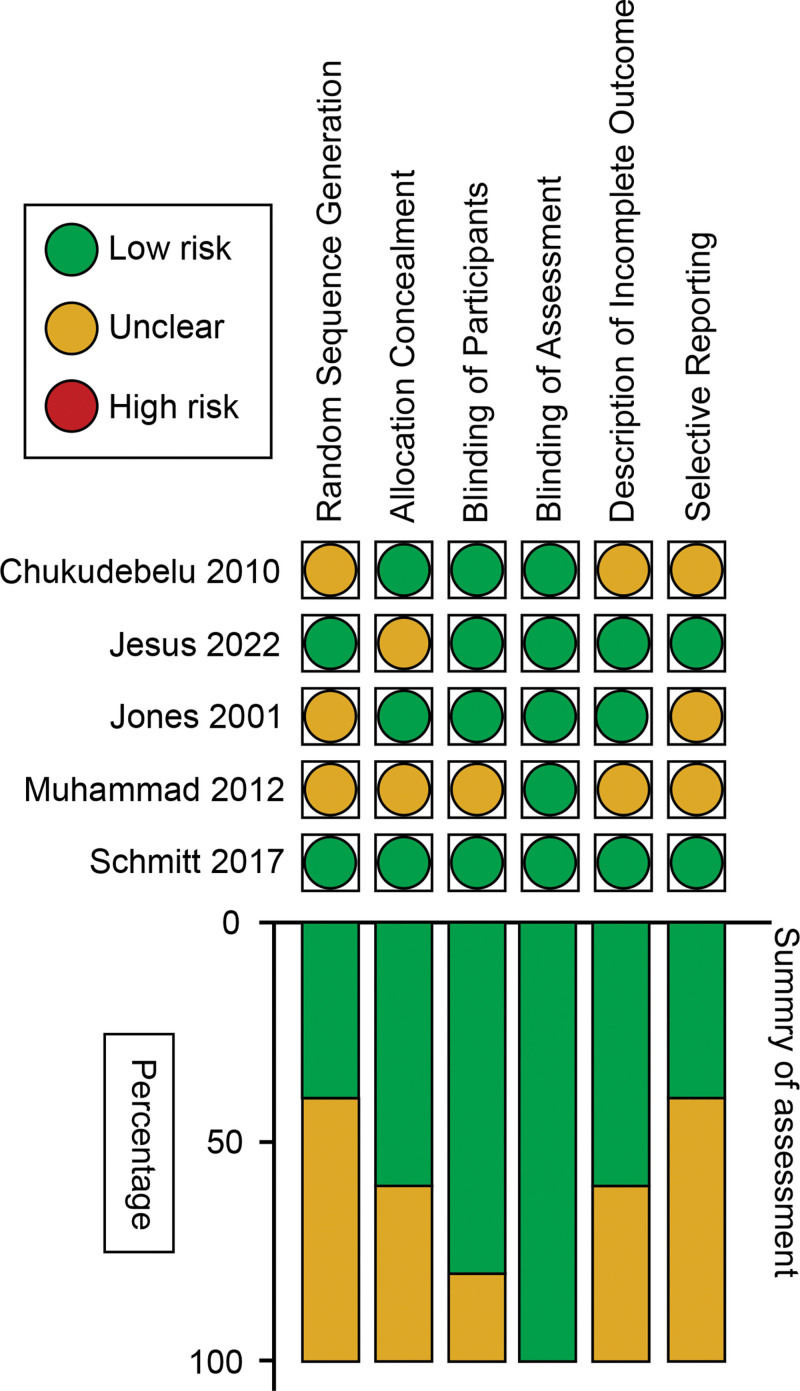
Bias assessment regarding each risk of bias item and summary of the included studies.

According to the risk item of bias, we noticed that only 2 of the included trials^[26,28]^ described random sequencing generation, and 3 of them^[25,26,28]^ reported allocated concealment. Four of them were designed as double blinding, and only 1 RCT^[27]^ failed to elucidate blinding to patients. Three trials^[20,26,28]^ described incomplete data and withdrawals, but only 2 studies^[26,28]^ clearly avoided the risk of selective reporting (Fig. 2). Along with these risks of bias in each study, each of them was rated by the Jadad scoring system; 4 of them^[20,25,26,28]^ were considered high-quality, and only 1^[27]^ was regarded as low-quality due to its unclear description of the methodological process (Table 2).

**Table 2:** The Jadad Scoring of included RCTs.

Author	Year	Item							Score	Quality
		Described “randomized” (1/0)	Appropriate randomization (1/0)	Described “double blind” (1/0)	Appropriate blinding (1/0)	Description of withdrawals (1/0)	Inappropriate randomization (0/−1)	Inappropriate blinding (0/−1)		
Chukudebelu	2010	1	0	1	1	0	0	0	3	High
Jesus	2022	1	1	1	1	1	0	0	5	High
Jones	2011	1	0	1	1	1	0	0	4	High
Muhammad	2012	1	0	0	0	0	0	0	1	Low
Schmitt	2017	1	1	1	1	1	0	0	5	High

### 3.3. The effects of gastric aspiration on reducing postoperative vomiting

**Figure 3.**
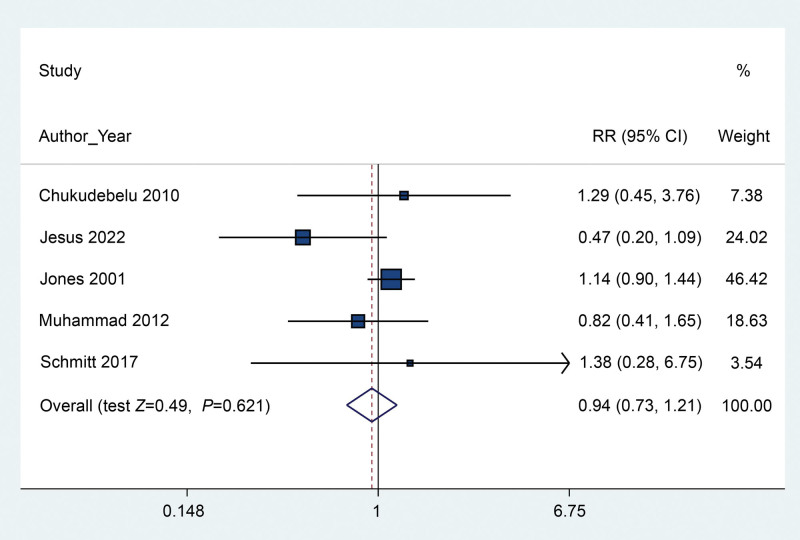
Forest plot between the packing and no packing groups regarding the incidence of vomiting.

**Figure 4.**
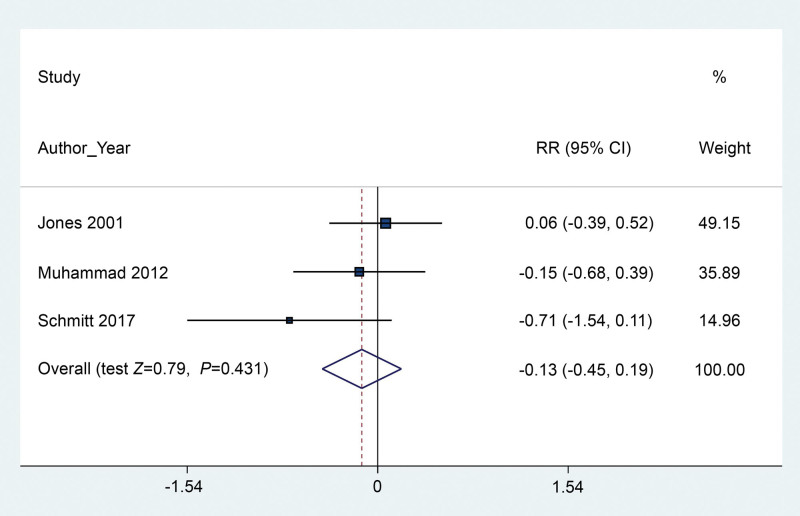
Forest plot between the packing and no packing groups with respect to the number of episodes of vomiting.

**Figure 5.**
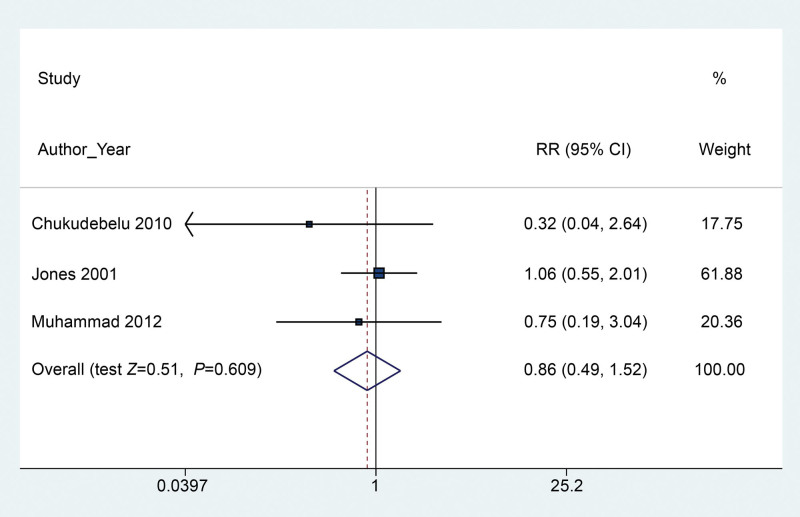
Forest plot between the packing and no packing groups with respect to the frequency of antiemetics use.

We pooled the estimated incidence of vomiting based on all 5 included RCTs by quantitative calculation of RR and associated 95% CI using meta-analysis. The results indicated that the application of gastric aspiration failed to reduce the risk of postoperative emesis (RR 0.94, 95% CI 0.73–1.21) (*P* = .621) (Fig. 3) with low heterogeneity (*I*^2^ = 32%). Three trials containing 152 participants reported the parametric data of the number of episodes of POV. The results of the pooled estimation revealed no significant difference between the gastric aspiration group and the no gastric aspiration group (SMD −0.13, 95% CI −0.45–0.19) (*P* = .431) (Fig. 4), with low heterogeneity (*I*^2^ = 23.6%). For the frequency of antiemetic use, 3 trials were included in the pooled estimation, and the results indicated that the application of rescue antiemetics was not decreased even when gastric aspiration was used (RR 0.86, 95% CI 0.49–1.52) (*P* = .609) (Fig. 5) based on a fixed-effects model (*I*^2^ = 0.0%).

### 3.4. Publication bias and recommendation of evidence

Although the majority of included trials were defined as high-quality and no obvious risk of bias was detected, we evaluated publication bias by funnel plots and Egger’s test. No obvious asymmetry was noticed with testing with a funnel plot regarding each outcome (Supplementary Figure S1, Supplemental Digital Content 1, http://links.lww.com/MD/L350). Meanwhile, the Egger test also demonstrated that no significant publication bias existed in the current study (Supplementary Figure S2, Supplemental Digital Content 1, http://links.lww.com/MD/L351). Considering all these results and the design of the included trials, we assessed the level of evidence regarding each outcome according to the GRADE rating. Imprecision was observed in each outcome. Moreover, the limitations of design, such as insufficient calculation of sample size and unclear description of random generation, were also important factors that degraded the level of evidence. Overall, the recommendation of evidence in the current study was rated low (Table 3).

**Table 3:** The quality and recommendation of the evidence according to the GRADE system.

Outcomes	Anticipated absolute effects (95% CI)*		Relative effect (95% CI)	No. of participants (studies)	Quality of the evidence (GRADE)**
	Assumed risk (control group)	Corresponding risk (aspiration group)			
Incidence of vomiting	387 per 1000	364 per 1000 (283–468)	RR 0.94 (0.73–1.21)	274 (5 RCTs)	⊕⊕Low^1^^,^ 2
Episode of vomiting	The mean episode is 1.99	The mean episode is 0.13 lower (−0.45–0.19)	–	152 (3 RCTs)	⊕⊕Low^1^^,^ 2
Application of rescue antiemetics	216 per 1000	344 per 1000 (292–407)	RR 0.86 (0.49–1.52)	167 (3 RCTs)	⊕⊕Low^1^^,^ 2

High-quality (⊕⊕⊕⊕): Further research is very unlikely to change our confidence in the estimate of effect

Moderate quality (⊕⊕⊕): Further research is likely to have an important impact on our confidence in the estimate of effect and may change the estimate

Low-quality (⊕⊕): Further research is very likely to have an important impact on our confidence in the estimate of effect and is likely to change the estimate

Very low-quality (⊕): We are very uncertain about the estimate

1 The confidence intervals overlapped the line of no effect (−1)

2 Lacking calculation of the sample size and test power (−1)

* The basis for the assumed risk is the mean control group risk across studies. The corresponding risk (and its 95% confidence interval) is based on the assumed risk in the comparison group and the relative effect of the intervention (and its 95% CI)

** GRADE Working Group grades of evidence

## 4. Discussion

The current meta-analysis included 5 RCTs containing 274 participants to evaluate the clinical effects of gastric aspiration on ameliorating postoperative vomiting. After quantitative pooled estimation, our results revealed no significant difference between the gastric aspiration group and the no gastric aspiration group regarding the incidence and the number of episodes of POV and the prevalence of rescue antiemetic use. The majority of included trials were rated as high-quality, and no obvious bias was detected; however, the overall recommendation of evidence was low due to the defects of design in certain included studies.

The mechanism of postoperative emesis is complex. The basis of vomiting is based on the afferent and efferent nerve axis connected and monitored by the medulla oblongata, which is the center that controls vomiting.^[25,29]^ Hence, the risk factors that may stimulate the nerve axis, such as tobacco or drug abuse, gastroparesis, application of narcotic or analgesic drugs, surgical type and procedures, could be potential causes of POV.^[30–33]^ In other words, the risk factors for POV included preoperative patient factors and intraoperative surgical and anesthetic factors. Omitting the uncontrolled patient factors, with the same anesthetic process, the elimination of surgical risk factors was the optimal way for surgeons to reduce POV. For oral and maxillofacial surgery, such as tonsillectomy and bimaxillary osteotomy, intraoperative surgical debris, secretions and blood inevitably enter the stomach and increase the gastric content and pressure. The swallowed blood and surgical manipulation were found to trigger chemoreceptors and mechanoreceptors in the oropharynx and stomach.^[6,27,34]^ Thus, swallowed surgical blood was hypothesized to be a strong peripheral emetic stimulus.^[3,6,11,28]^ Therefore, the application of gastric aspiration was mainly for evacuating the gas, fluid and other intragastric mixtures to lessen the surgical stimulation and reduce the possibility of POV.

In the literature, Jesus et al^[26]^ reported the first RCT that demonstrated that gastric aspiration was effective in reducing POV after orthognathic surgery and consequently decreasing inpatient period and hospitalization costs. Moreover, a recent retrospective study with a large-scale sample also determined its effects on reducing the incidence of POV and operation time in orthognathic surgery.^[19]^ They declared that the clinical benefits of gastric aspiration were not only reflected in reducing POV but also may prevent POV from the original source, such as reducing the operation time. Another clinical observational investigation also supported the utilization of gastric aspiration in ear, nose, and throat surgery because of the lower incidence and severity of PONV after gastric aspiration application.^[33]^ However, more clinical trials may deny the clinical benefits of gastric aspiration in this field. Jones et al^[20]^ conducted the first traceable RCT which showed a negative impact on reducing POV, surgical blood loss, and hospitalization stay. Two other RCTs also investigated the effectiveness of gastric decompression in tonsillectomy for the alleviation of postoperative emesis, but no positive result was discovered.^[25,27]^ For orthognathic surgery, Schmitt et al^[28]^ also denied the potential benefit of gastric aspiration based on a small sample RCT. According to our results, gastric aspiration revealed no significant clinical benefit regarding the incidence and number of episodes of POV, which was consistent with the majority of published RCTs. Therefore, we preliminarily concluded that gastric decompression should not be recommended for alleviating POV.

Notably, the current study also determined no obvious difference between the gastric aspiration group and the no gastric aspiration group with respect to the application rate of rescue antiemetics. As mentioned above, vomiting behavior is based on the nerve reflex axis and reflex center. The antiemetic agent was theoretically composed of neurotransmitter antagonists acting in the periphery or/and the nerve center.^[25,35,36]^ Antiemetics were routinely applied during anesthesia for prophylaxis of POV and utilized for postoperative rescue. In the included trials, different antiemetic agents were applied, although postoperative rescue antiemetics were basically used for symptomatic treatment. The impact of different antiemetic agents on antiemetic use is currently not fully understood. More importantly, the impact of postoperative antiemetic type on the efficacy of gastric aspiration is also needed in future clinical research.

Gastric aspiration is a nonpharmacological strategy for the prevention of POV. Theoretically, gastric decompression and evacuation of gastric content mixed with swallowed blood could obviously reduce the risk of POV by eliminating a peripheral emetic stimulator. However, the results of the current meta-analysis revealed that gastric decompression had no benefits in relieving POV regarding the relative 3 outcomes. According to the purpose and theory, gastric decompression was a prerequisite for ameliorating POV. However, at the same time, indwelling gastric tubes may also stimulate the pharynx and larynx, although they are not a continuous catheterization in oral and maxillofacial surgery.^[19]^ In addition, the method of gastric tube insertion could be another factor influencing POV. Erkalp et al argued that the orogastric method would be more effective than the nasogastric method due to easier and fewer pharyngology-related complications, which may be a potential interfering factor of POV.^[33]^ Therefore, the association of tube stimulation, insertion method and POV could be another interesting clinical research direction.

To the best of our knowledge, the current study is the first meta-analysis investigating the clinical effects of gastric decompression in oral and maxillofacial surgery. We found that gastric aspiration revealed no clinical benefits for the amelioration of POV according to our quantitative results. Nevertheless, some inevitable defects also need to be addressed. First, our quantitative analysis was based on RCTs, but only 5 eligible trials were included. The total sample size may be insufficient for convincing conclusions regarding the outcomes. Notably, observational studies with larger scales exhibited different conclusions compared with ours.^[19,33]^ Although we excluded nonRCTs due to their low-quality, we still need to draw conclusions with caution. Moreover, the included trials contained multiple confounding factors, such as subspecialty surgical procedures, and subgroups could not be performed due to insufficient comparisons. Additionally, for all the outcomes, the recommendations of evidence were not convincing enough because of the confounding factors, which may interfere with clinical decision-making.

In summary, our research quantitatively determined that gastric aspiration revealed no benefit in ameliorating POV in oral and maxillofacial surgery. Gastric decompression was not recommended for patients who underwent oral and maxillofacial procedures. On the other hand, we realize that the overall sample size of the current study was not high enough and should be further studied in the future, although the majority of included trials were high-quality and no obvious bias was detected. However, more high-quality large-scale RCTs are still needed.

## Author contributions

TG designed the research. XZ, XX, MS, YY and ZF performed the research and data collection. XZ, XX, MS, YY and JY contributed analytic tools and data analysis. JY and TG wrote the paper. All the authors have read and approved the final manuscript.

Data curation: Xushu Zhang, Yao Yao, Jian Yang.

Investigation: Xushu Zhang, Xiaojuan Xie, Yao Yao, Zhen Feng.

Software: Xushu Zhang, Xiaojuan Xie, Yao Yao.

Methodology: Xiaojuan Xie.

Conceptualization: Min Shi, Tao Guo.

Writing—original draft: Min Shi, Jian Yang, Tao Guo.

Formal analysis: Yao Yao.

Resources: Jian Yang.

Writing—review & editing: Jian Yang, Tao Guo.

Supervision: Tao Guo.

## Supplementary Material






