# Supplementary material for: The efficacy of gastric aspiration in reducing postoperative vomiting after oral and maxillofacial surgery: A meta-analysis
Source: Medicine (Baltimore). 2024 Feb 16;103(7):e37106. doi: 10.1097/MD.0000000000037106 (PMC10869046; doi:10.1097/MD.0000000000037106)
Supplement: Supplementary file 1 [file medi-103-e37106-s001.doc]

**Table S1.** Example of search order in Pubmed.

| Order | MeSH |
| --- | --- |
| 1 | gastric aspiration |
| 2 | gastric negative pressure |
| 3 | gastric decompression |
| 4 | 1 OR 2 OR 3 |
| 5 | oral |
| 6 | dental |
| 7 | orthognathic |
| 8 | maxillofacial |
| 9 | mandibularis |
| 10 | facial plastic |
| 11 | 5 OR 6 OR 7 OR 8 OR 9 OR 10 |
| 12 | vomit |
| 13 | vomiting |
| 14 | emesis |
| 15 | 12 OR 13 OR 14 |
| 16 | 4 AND 11 AND 15 |
| 17 | randomized |
| 18 | controlled clinical trial |
| 19 | randomized controlled trial |
| 20 | retrospective |
| 21 | observational |
| 22 | cross-sectional |
| 23 | 17 OR 18 OR 19 |
| 24 | 20 OR 21 OR 22 |
| 25 | 23 NOT 24 |
| 26 | 16 AND 25 |
