# Supplementary figures and images for: The efficacy of gastric aspiration in reducing postoperative vomiting after oral and maxillofacial surgery: A meta-analysis
Source: Medicine (Baltimore). 2024 Feb 16;103(7):e37106. doi: 10.1097/MD.0000000000037106 (PMC10869046; doi:10.1097/MD.0000000000037106)

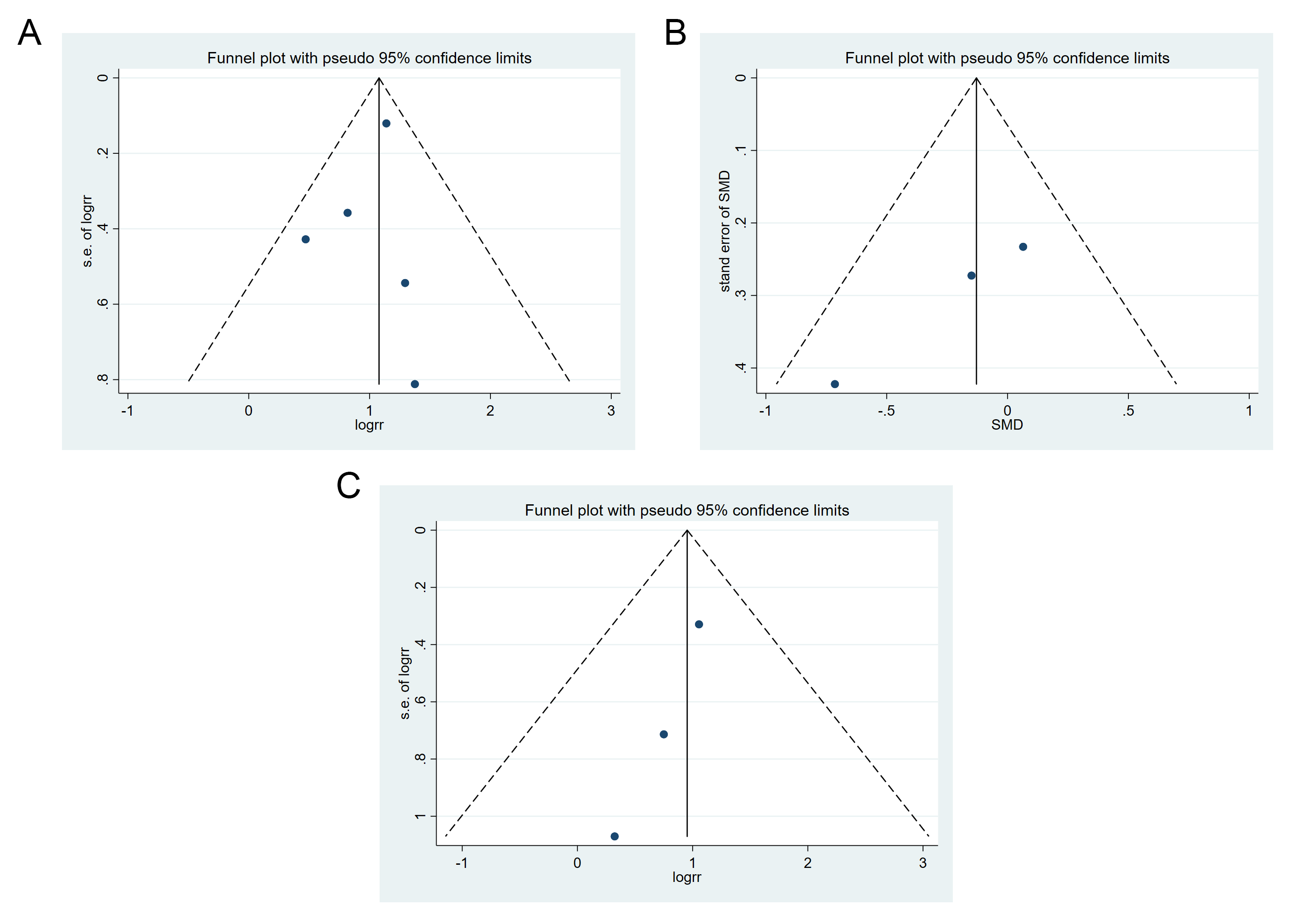

Supplement: Supplementary file 2 [file medi-103-e37106-s002.tif]

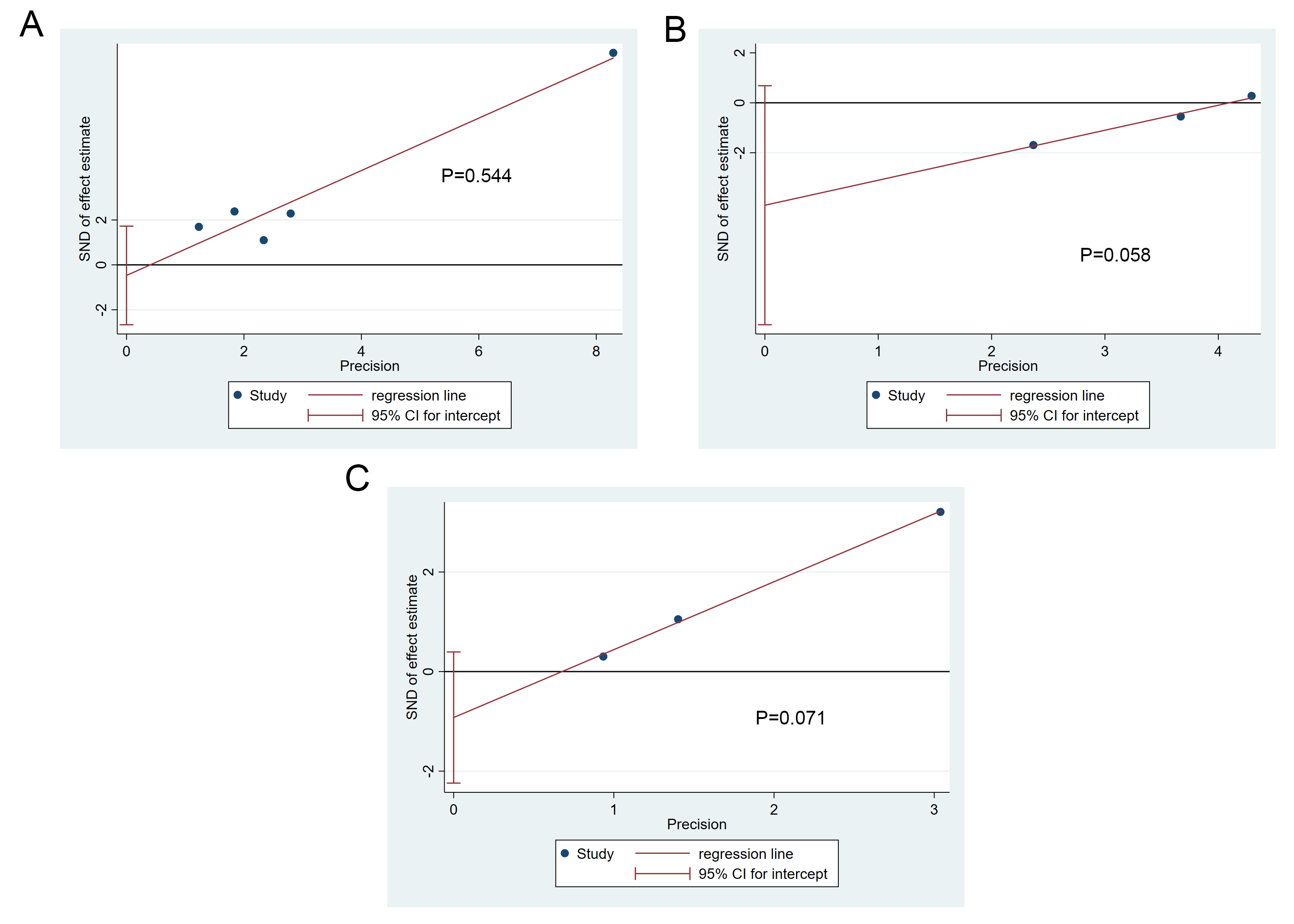

Supplement: Supplementary file 3 [file medi-103-e37106-s003.tif]
